# The Initial Assessment and Management of Cervical Spine Injuries: A Comprehensive Review

**DOI:** 10.7759/cureus.88805

**Published:** 2025-07-26

**Authors:** Nur Amelia S Shaharudin, Olivia A Dunseath, Nur Aina Azmi, Ning Yee Aun

**Affiliations:** 1 Trauma and Orthopaedics, Barts Health NHS Trust, London, GBR; 2 Emergency Department, Rotherham General Hospital, Sheffield, GBR; 3 Psychiatry, Hertfordshire Partnership University Foundation Trust, Hertfordshire, GBR

**Keywords:** advanced trauma life support (atls), axis fracture: cervical spine trauma, cervical spine fracture, review of literature, spine and trauma, spine imaging

## Abstract

Cervical spine injuries (CSIs) are the most frequently encountered spinal injuries resulting from blunt trauma, owing to the cervical region’s anatomical vulnerability and high mobility. Early recognition and spinal motion restriction (SMR) are essential in preventing secondary spinal cord injury (SCI), particularly in pre-hospital settings. This literature review explores the comprehensive management of suspected CSIs from the pre-hospital setting to definitive care in the hospital. It highlights the importance of clinical suspicion based on the mechanism of injury, appropriate application of SMR, and the role of imaging modalities such as CT and MRI in diagnosis. Clinical decision tools, including the National Emergency X-Radiography Utilization Study (NEXUS) criteria and Canadian C-Spine Rules (CCR), are discussed in relation to reducing unnecessary imaging while maintaining patient safety. The review also addresses current debates around cervical collar use, the necessity of structured neurological assessments, and the importance of multidisciplinary collaboration. Classification systems such as the Subaxial Cervical Spine Injury Classification System (SLIC) and AO Spine Trauma Classification are evaluated for their role in guiding treatment decisions. Effective care for CSI requires timely intervention, accurate imaging, and coordination among emergency, radiology, intensive care, and spinal surgical teams to minimise neurological complications and improve outcomes.

## Introduction and background

The cervical spine's anatomical structure, characterised by its high mobility and exposure, makes it the most injury-prone region of the spine [1,2]. This susceptibility is further exacerbated by blunt trauma, which can subject the cervical spine to injury from axial loading, flexion, extension, rotation, lateral bending, and distraction [1,3].

Vehicular trauma and unintentional falls are the major contributors to spinal cord injury (SCI) [1,4]. Its management is further complicated in the older population as they are more susceptible to falls and underlying comorbidities such as arthritis and osteoporosis [4,5]. Due to its unique anatomy, cervical spine injuries (CSIs) require a high degree of clinical suspicion from clinicians in the pre-hospital setting. CSIs are commonly classified as either stable or unstable. Stable injuries generally pose a lower risk of significant deformity or neurological damage and can often be managed conservatively. In contrast, unstable CSIs, if not already resulting in spinal cord damage from the primary trauma, can lead to further SCI, also known as secondary SCI. Secondary SCI arises as a pathophysiological response to the initial trauma and may persist over a prolonged period. Early spinal motion restriction (SMR) is crucial until further assessment and appropriate imaging protocols enable a proper diagnosis and prompt management of injuries.

Patients with suspected CSI can present with polytrauma with varying levels of consciousness, which can cause challenges for clinicians. A structured approach, such as the Advanced Trauma Life Support (ATLS) protocol [1], must be applied when assessing patients, along with the timely use of appropriate imaging modalities like CT and MRI, which are crucial for accurate diagnosis. Both initial and definitive management require input from multiple specialties to prevent further deterioration and minimise the risk of SCI. This literature provides an overview of the initial assessment and management of patients with suspected CSIs.

## Review

Pre-hospital management

The anatomy of the cervical spine is commonly divided into the upper and lower cervical spines. The upper cervical spine consists of C1 and C2, also known as the atlas and axis, and the rest of the cervical spine is considered the lower cervical spine (C3-C7). Among the most commonly encountered fractures are odontoid fractures of C2 and subaxial compression fractures involving the lower cervical vertebrae. Due to the wide nature of the cervical canal from the foramen magnum to the lower part of C2, on-scene mortality rates are approximately one-third of patients with injuries in this region [1]. A key factor in upper cervical spine stability is ligamentous integrity [2]. For the lower cervical spine, SCIs are more commonly due to vertebral column injuries [1].

Understanding the anatomy helps in determining whether or not a surgical intervention is necessary in CSIs; however, management of such injuries begins on-site in the pre-hospital setting. Spinal injury can often be suspected in the pre-hospital setting based on the mechanism of trauma, particularly in cases of blunt trauma, as outlined in both the British Orthopaedic Association Standards for Trauma (BOAST) guidelines and ATLS principles [1,6].

CSIs cannot be excluded from a lack of neurological deficit alone. Due to this, SMR must be applied from on scene when indicated to prevent excessive mobility that can lead to further injuries, especially secondary spinal cord traumas. ATLS principles recommend the use of a cervical collar to assist in protecting the cervical spine [1]. SMR involves positioning the patient on a firm, liftable surface, such as a long backboard or a scoop stretcher, together with a fitted cervical collar and head blocks. This is also known as ‘triple immobilisation’. The purpose of SMR is to minimise excessive movement while maintaining neutral spine alignment. Full spinal immobilisation is carried out to reduce the risk of noncontiguous spinal injuries [4]. Although patient transfer poses a risk of displacing an unstable spinal injury, SMR helps to minimise this risk [7].

Indications for SMR following blunt trauma include an acute altered level of consciousness (ie: intoxication, reduced Glasgow Coma Score), midline neck or back pain with or without tenderness, focal neurological signs and/or symptoms, anatomic deformity of the spine, and presence of distracting circumstances (ie: long bone fractures, crushing injuries, burns) or injury that potentially impair the patient’s ability to engage in a reliable examination [4,8]. Notably, the BOAST guidelines discourage continuing spinal precautions for more than 48 hours, including practices such as log rolling for mobilisation, partly due to the associated increased risk of pressure sores [6]. This highlights that, while spinal immobilisation is crucial in the initial management, prompt diagnosis and definitive treatment upon arrival at the hospital are equally important.

The use of cervical collars has been the subject of considerable debate in recent years regarding their efficacy. While the ATLS guidelines play a significant role in the management of trauma patients, there is currently limited evidence to support the benefits of collar use outweigh its associated risks [9,10]. An immobilisation strategy utilising head blocks and straps on a spine board or scoop stretcher, while omitting a cervical collar, is often practised for this reason. Reported risks of collar use include pain, raised intracranial pressure, pressure ulcers, restricted clinical examination, delayed hospital transfer, airway management difficulties, and fracture displacement in older patients [9-12]. Regardless of collar use, the cervical spine should be immobilised, and the patient promptly transferred to the hospital.

Initial hospital assessment

On arrival at the hospital, a trained team should ideally be available for patient transfer and assessment. A trauma call is often activated in advance to ensure proper preparation. In suspected CSIs, both inadequate SMR and delayed diagnosis can lead to serious neurological damage [4]. A clear and concise handover between pre-hospital and hospital staff is crucial to ensure the mechanism of injury, key clinical findings, and pre-hospital interventions are communicated. This is followed by patient assessment using the ATLS protocol, starting with airway management while maintaining cervical spine stabilisation. ATLS recommends maintaining triple immobilisation, but as previously highlighted, some prefer stabilisation without a collar. Patient assessment is guided by their level of consciousness-whether awake or obtunded. In those with reduced consciousness or a GCS <8, should raise suspicion for head injury, making securing a definitive airway essential.

The next step in the ATLS primary survey involves assessing breathing, followed by circulation, disability, and exposure, ensuring they are evaluated in this sequence. In circulation, it is essential to recognise that neurogenic shock, commonly associated with SCI, can present with hypotension [1]. As the management of neurogenic shock differs from that of haemorrhagic shock, obtaining appropriate imaging as soon as it is safe to do so is essential. For patients over the age of 15, CT is the initial imaging modality of choice [13]. It is also important to distinguish neurogenic shock from spinal shock, as both may result from the same mechanism of injury. Spinal shock results in a transient loss of all spinal cord function (motor, sensory, reflexes) below the site of the SCI. In contrast, neurogenic shock is a haemodynamic disturbance caused by the disruption of the sympathetic nervous system control.

All unconscious patients or those with an altered mental state will require further imaging, typically CT or MRI of the head and cervical spine [1,3,4,9,13]. In awake patients, a detailed neurological assessment should be conducted promptly and thoroughly; however, this must not delay imaging. Such assessments should be meticulously documented, with best practice involving the use of standardised tools such as the International Standards for Neurological Classification of Spinal Cord Injury (ISNCSCI) clinical documentation tool, published by the American Spinal Injury Association (ASIA)-commonly referred to as the ASIA chart.

Although the absence of neurological symptoms does not necessarily indicate cervical spine clearance, not all patients with a suspected CSI require imaging. The need for radiological assessment can be determined using one of two established criteria: the National Emergency X-Radiography Utilization Study Group (NEXUS) and Canadian C-Spine Rules (CCR). It is important to note that only around 3% of cervical trauma patients have findings that are significant clinically, and a larger proportion, unfortunately, are being exposed to unnecessary radiation [14-16]. The NEXUS and CCR criteria help identify clinically significant CSI and are useful tools that help prevent unnecessary imaging in patients [4].

However, these criteria should not be applied to adults over 65 years of age or individuals with conditions that result in a rigid spine (e.g., ankylosing spondylitis), as these populations are at higher risk of severe injuries from low-energy trauma. In such cases, detailed imaging of the cervical spine is essential [13]. Interestingly, in a meta-analysis carried out by Vazirizadeh-Mahabadi et al. CCR has a better screen accuracy compared to NEXUS [14].

Initial management of CSI

CT findings are crucial in guiding management and determining the urgency of management, with the primary aim being cervical spine stabilisation. A decision must be made whether to maintain SMR or switch to a different brace (e.g., Miami-J), based on imaging and input from the spinal team. If the cervical CT is negative, especially in awake patients, the spine can be cleared, and immobilisation discontinued, provided there are no other injuries [4]. However, due to the limitations in detecting soft tissue injuries in CT, patients with high suspicion of CSI should be discussed with spinal services, as MRI may be necessary to assess ligamentous integrity [1-4,16,17].

Unstable cervical spine fractures or injuries can result in SCI. SCIs are typically classified into primary and secondary types. Primary SCI occurs due to mechanical forces that directly damage the spinal cord, whereas secondary SCI refers to the subsequent neurological injuries that result from the initial trauma, which can progress over a prolonged period [18]. In patients with neurological findings indicative of SCI during the initial assessment, the primary goal of management is to prevent the progression of secondary SCI [4]. Therefore, should the unstable cervical spine fracture be appropriate for surgical intervention, time is of the essence [4].

In addition to surgical intervention, pharmacological strategies also play a role in the acute management of SCI. Effective analgesia is also an important element of the initial management of any fractures, including cervical spine fractures. NICE recommends the use of intravenous morphine first-line, and intranasal diamorphine or ketamine as second line [19]. It is well documented that chronic neuropathic pain is a known complication of SCI, occurring in up to 40% of cases and significantly affecting patients’ quality of life [19,20]. Despite so, there is a lack of evidence that supports pre-emptive administration of analgesia targeting neuropathic pain [19]. The administration of high-dose steroids as a neuroprotective measure is common in practice. However, their routine use remains controversial, as current evidence indicates no significant benefit in neurological improvement [21]. Often, patients with SCIs require intensive care unit (ICU) admission for continuous monitoring, as respiratory compromise within the first five days post-injury, due to ascending oedema, is a leading cause of mortality [3,22-24]. Additionally, neurogenic shock, commonly associated with SCIs, can impair spinal cord perfusion, resulting in secondary injury if not recognised promptly. Managing this condition with vasopressors, inotropic support, and mean arterial pressure (MAP) monitoring also necessitates ICU care [25].

Definitive management of CSI

It is important to understand that the primary goal of definitive management for a CSI is to minimise further damage, thereby preventing an increase in functional disability [3]. Definitive management can be either through surgical or conservative measures. These decisions are made with input from the spinal team, along with other relevant specialties, if the patient has polytrauma injuries. There are a number of scoring systems used as adjuncts to help decide on the appropriate intervention. One classification system used is the Cervical Subaxial Injury Classification System (SLIC). This points-based system looks at the injuries to the subaxial cervical spine (C3-C7) and assesses fracture morphology, discoligamentous complex (DLC) integrity, and neurological status of patients [4,26]. This scoring system places a heavy reliance on the integrity of the DLC. However, while CT scans can provide some indication of DLC integrity, MRI remains the gold standard, and this reliance on MRI may delay the decision-making process for definitive management. It is important to highlight that the classification is limited to the lower cervical spine. 

A more recent but also widely used classification system is the AO Spine Trauma Classification, which categorises spinal injuries into four distinct regions: the upper cervical spine, subaxial cervical spine, thoracolumbar spine, and sacrum. This system assesses injury morphology, neurological status, and patient-specific modifiers to generate a severity score, which helps guide decision-making for definitive management, be it conservative or surgical, through a structured algorithm [4,27,28]. Interestingly, as this classification utilises CT scan, which helps reduce global inequalities in access to care as they are more readily accessible, the lack of soft tissue imaging, which is better supported via MRI, especially when it comes to ligamentous injury, might put it at a disadvantage, especially when assessing the upper cervical spine. All in all, both classification systems provide a structured approach in decision making for surgeons, but the severity scores do not always clearly dictate clear guidance for management; clinicians must rely on their clinical judgement to determine whether surgery is necessary and its acuity. While current systems have limitations, future advancements will likely address these shortcomings and enhance patient care, as timely management is essential in preventing secondary SCIs.

## Conclusions

The cervical spine is the most frequently affected region of the spine in cases of blunt trauma. The management of CSIs begins in the pre-hospital setting with early suspicion and SMR. For patients who survive hospital transfer, prompt and thorough assessment, imaging, and timely intervention are crucial to prevent further injury. The ATLS framework provides a structured approach to primary assessment, which has been widely adopted by clinicians and trauma centres worldwide. A systematic approach, supported by adjuncts such as the NEXUS criteria and CCR, plays an important role in guiding the selection of subsequent imaging modalities to confirm or rule out diagnoses. A multidisciplinary approach involving specialities such as emergency medicine, radiology, intensive care, and spinal services enables the patient to receive appropriate initial and definitive management for their injuries.
